# Early onset of breast cancer in Black British women: how reliable are the findings?

**DOI:** 10.1038/sj.bjc.6604625

**Published:** 2008-09-09

**Authors:** J D Ingleby

**Affiliations:** 1 Department of Interdisciplinary Social Science, Faculty of Social and Behavioural Sciences, Utrecht University, P.O. Box 80.140, 3508 TC Utrecht, Netherlands

**Sir,**

The article by Bowen *et al* (2008a), reporting that Black women in Hackney presented for breast cancer at a median age of 21 years younger than that of White women and had more serious pathology, has given rise to widespread concern and a petition to 10 Downing Street calling for a programme of early breast cancer screening for Black women. If the conclusions of the article are correct, the refusal of such a screening programme would amount to no less than a racial injustice. If they are not correct, its introduction would lead to unnecessary anxiety, discomfort and expense. All the more reason, then, to require from such a study the highest standards of scientific rigour.

Two letters to the editor (Dindyal *et al*, 2008 and Cichowska *et al*, 2008) have raised concerns about this study. The authors' replies to these letters (Bowen *et al*, 2008b, 2008c) did not seem to me to deal with these points adequately, and I therefore subjected their published data to further analysis. The results indicate that it may be premature to conclude that Black women in general have a higher risk of contracting early breast cancer. The risk would seem to be confined to one ethnic subcategory, ‘Black Other’. In this category, however, the risk seems to be very high.

Dindyal *et al* (2008) and Cichowska *et al* (2008) emphasised the importance of distinguishing among Black women originating from different geographical regions. In their reply to Dindyal *et al* (2008), the authors present a breakdown of the sample of Black patients according to their ethnic subcategory. However, this does not answer the question of whether there are significant differences among these groups. To find out, we would need to compare the numbers of patients in each subcategory with the size of the underlying population. This information is readily available from the excellent website maintained by the Office for National Statistics (www.nomisweb.co.uk).

A slight problem arises in performing these calculations because the ethnic subcategories used by Bowen *et al* (2008a) are not all labelled in the same way as in the census. They classify their patients as ‘Black African, African Caribbean, Black British or Black Other’, whereas the census data refer to ‘Black or Black British’, subdivided into ‘Black African’, ‘Black Caribbean’ and ‘Other’. The four patients in the authors' category ‘Black British’ can be allocated to the census category ‘Other’, as 83% of this group are British-born. I have assumed that the authors' category ‘African Caribbean’ corresponds to the census category ‘Black Caribbean’, as it is hard to see what else it could mean.

The results of this analysis were surprising. First, although the total Black population of Hackney reported by the authors was approximately the same as that reported in the census data, their figure for the White population of interest was 34% higher than in the census. This seemed to be because they had included the category ‘White Other’ in their population estimate, whereas they had *excluded* this category from their patient sample.

In fact, Bowen *et al* (2008a) give contradictory information about the inclusion criteria for their White patients. In their article, they state that ‘data were obtained from … 191 White British women’, but in their reply to Dindyal *et al* (2008), they say ‘all White women included reported themselves to be White English, Welsh or Scottish *or White Irish*’ (my italics). I have assumed that the second statement is more accurate and that White Irish patients were included.

Excluding persons under 20 from the population totals, as the risk of contracting breast cancer at such an early age is negligible, Table 1 shows the overall incidence rates for each group over a 10-year period.

Comparing the White and Black Other groups, the odds ratio (OR) is 3.43 (*χ*^2^=36.1; *P*<0.0001). However, no significant difference is found between the White group and any of the remaining Black groups. The data also need to be adjusted for differences in socioeconomic status, which only the authors are in a position to do. (It would be interesting to know what influence this variable has: unfortunately no information is given on this point.) But these figures suggest strongly that only the ‘Black Other’ group has an increased overall risk of breast cancer. In this small group (2.5% of the total population), the increase is indeed dramatic and needs to be investigated urgently.

Of course, Bowen *et al* (2008a) are not concerned with overall rates but with the age of onset and clinical features of breast cancer. There was a striking difference of 21 years between Black and White women in the median age of presentation. However, as Cichowska *et al* (2008) point out, this figure takes no account of possible differences in the age structure of the Black and White populations. According to the authors, no such differences were significant; furthermore, they say that they were unable to calculate detailed age-specific rates, because information about the age structure of the population was only available in three broad age groups (0–15, 16–59 and 60 years or more).

Both statements are puzzling. The difference in age structure among the groups as reported by the authors in their Table 1 is, in fact, highly significant (*χ*^2^=374.6; *P*<0.0001). Second, detailed information about the age structure of ethnic groups is readily available in the census data for Hackney. By combining Table 4 of the authors' original article with this data, it was possible to calculate age-specific incidence rates, although I could not perform this analysis for the ethnic subgroups separately or adjust for socioeconomic status. Figure 1 shows the results.

Even after allowing in this way for the different age structure of the two groups, incidence rates below the age of 50 years were significantly higher in the Black group (age 20–39 years, OR=4.41, *χ*^2^=14.8, *P*<0.0002; age 40–49 years, OR=1.95, *χ*^2^=8.0, *P*<0.005; other differences not significant. When carrying out five tests simultaneously, the Bonferroni correction requires an *α* of 0.01 instead of 0.05). These results support the authors' conclusions, although adjustment for differences in socioeconomic status might change the picture to some extent. Nevertheless, the overall figures quoted above suggest that these effects are mainly due to the category ‘Black Other’.

With the help of the census data, it is also possible to calculate how many patients would be expected in the Black group if it had the same age structure as the White group. The difference in median age would then fall to approximately 12 years: in other words, almost half of the difference reported by Bowen *et al* (2008a) is simply a consequence of the fact that there are relatively few Black women over the age of 60 in Hackney. It is regrettable that the figure of 21 years has received so much publicity in the media.

These results show clearly that it does not make sense, either in research or screening policy, to treat Black women as a single group. Moreover, looking at the variations in incidence rates within different Black groups suggests that *country of birth* may be an important variable.

As McCormack *et al* (2008) point out, ‘breast cancer incidence rates vary six-fold between industrialised and less-developed countries, and migrants from low-risk countries to high-risk countries have an intermediate risk’. On this basis, we would expect a lower incidence in the Black subgroups containing a higher percentage of foreign-born migrants. As Table 2 shows, this relationship holds within this study, suggesting that future studies should pay attention not only to self-reported ethnicity, but also to place of birth.

It should be borne in mind that data obtained from studies such as this one can never be entirely reliable, for the simple reason that the boundaries of local authorities are permeable. For various reasons, people may seek treatment at a hospital outside their own area. In this study, it is impossible to know how many women from Hackney obtained treatment at other hospitals, and the authors do not tell us how many of the patients in this study came from other areas. Moreover, census data are collected only once in 10 years, whereas populations can change rapidly.

The comparison of pathological and tumour features yielded hardly any indications of a Black/White difference: of the nine variables studied, only two showed significant group differences. It is not reported whether a Bonferroni or similar correction was used – but if this was not the case, none of the reported differences are significant, as with nine tests the Bonferroni correction requires an *α* of 0.005 instead of 0.05.

To sum up: in view of the concern their study has given rise to, it would seem highly advisable for the authors to reanalyse their data, taking care to match their categories as accurately as possible with those used in the census data and paying attention to the points raised by Dindyal *et al* (2008) and Cichowska *et al* (2008).

## Figures and Tables

**Figure 1:**
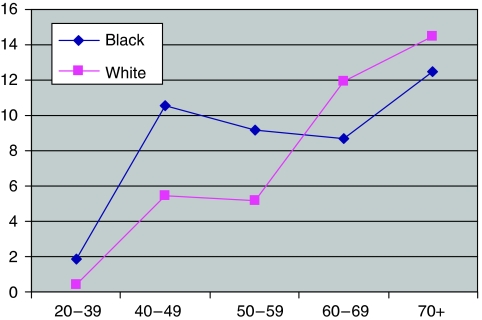
Age-specific incidence of breast cancer in the Black and White groups (patients per 1000 of the population).

**Table 1:** Overall incidence rates by ethnic subgroup (patients per 1000 of the population)

**Group**	**Incidence**
White British and Irish	4.9
Black Caribbean	5.5
Black African	3.6
Black Other	16.9
Black (total)	5.5

**Table 2:** Ethnic subgroup, % UK-born and overall incidence rates

**Subgroup**	**% Born in United Kingdom**	**Overall incidence**
Black African	37	3.6
Black Caribbean	50	5.5
Black Other	83	16.9
